# *CLAVATA* Was a Genetic Novelty for the Morphological Innovation of 3D Growth in Land Plants

**DOI:** 10.1016/j.cub.2020.06.015

**Published:** 2020-07-06

**Authors:** Chris D. Whitewoods, Joseph Cammarata, Zoe Nemec Venza, Stephanie Sang, Ashley D. Crook, Tsuyoshi Aoyama, Xiao Y. Wang, Manuel Waller, Yasuko Kamisugi, Andrew C. Cuming, Péter Szövényi, Zachary L. Nimchuk, Adrienne H.K. Roeder, Michael J. Scanlon, C. Jill Harrison

(Current Biology *28*, 2365–2376.e1–e5; August 6, 2018)

## Main Text

In this article, we characterized roles for CLAVATA in the development of a moss, *Physcomitrella patens*, focusing on the 2D to 3D growth transition. Ongoing work to further characterize mutant phenotypes identified some phenotype discrepancies among the *Ppclv1a* and *Ppclv1b* mutant lines published in the original paper. For this reason, we implemented further checks of the published manuscript and fully sequenced both *PpCLV1a* and *PpCLV1b* loci in all mutants originally reported in the Methods S1 figure “CRISPR/Cas9 strategy for generating *Ppclv1* mutants.” Although the conclusions of the paper remain valid, our investigations revealed errors that we wish to correct.

We found that *Ppclv1a* line 18 (Figures 4D and 4K; Methods S1, CRISPR figure, panel E) contained an 805 bp deletion at the *PpCLV1a* locus, but while *Ppclv1a* line 18 plants had phenotypes resembling WT plants, there was also a 9 bp deletion at *PpCLV1b*. We found no mutations at *PpCLV1a* or *PpCLV1b* in *Ppclv1a* line 29 or *Ppclv1a* line 32 (Methods S1, CRISPR figure, panels F and G), and these lines were indistinguishable from WT plants. The genotype of *Ppclv1b* line 2 (Figures 4E and 4L; Methods S1, CRISPR figure, panel H) was reported as a 2 bp deletion, but genome walking now confirms that there is a 4 bp deletion and >6 kb insertion at *PpCLV1b*, and the insertion comprises sequence integrated from the *pACT::Cas9* expression vector used to engineer the lines [1]. The genotypes of *Ppclv1b* line 9 and *Ppclv1b* line 33 (Methods S1, CRISPR figure, panels I and J) were not previously reported, and while *Ppclv1b* line 9 has a 47 bp deletion at the *PpCLV1b* locus, *Ppclv1b* line 33 has no mutation at *PpCLV1a* or *PpCLV1b*, and an indistinguishable phenotype from WT plants. The reported genotypes of *Ppclv1a1b* lines 6, 8, and 12 were accurate (Methods S1, CRISPR figure, panels K–M).

**Figure 1:**
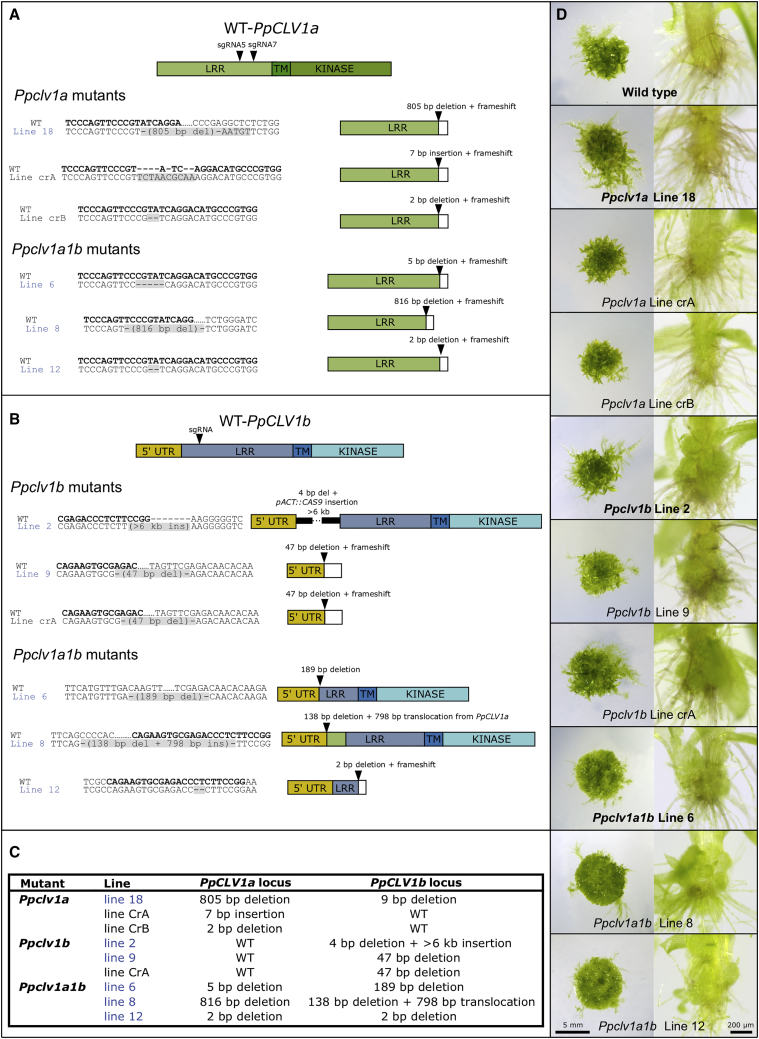
CRISPR/Cas9 Strategy for Generating *Ppclv1* Mutants (A) Strategy for mutagenizing *PpCLV1a* to generate *Ppclv1a* single mutants and *Ppclv1a1b* double mutants. Two guide RNAs were designed against the LRR domain of *PpCLV1a* and cloned into *pU3::Ppclv1a sgRNA5* and *pU3::Ppclv1a sgRNA7* expression vectors (original paper). Plants were co-transformed with *pU3::Ppclv1a sgRNA5*, *pU3::Ppclv1a sgRNA7*, *pNRF*, and *pACT::Cas9* (original paper), or with *pU3::Ppclv1a sgRNA7*, *pNRF*, and *pACT::Cas9* for the Correction. Sanger sequencing of full-length *PpCLV1a* and *PpCLV1b* loci in all lines verified the mutations illustrated. Sequences in bold represent sgRNA target sites on WT loci, gray boxes highlight mutations in each line, and schematics to the right illustrate the predicted effect of mutations on the protein. Lines with labels in blue were reported in the original paper. (B) Strategy for mutagenizing *PpCLV1b* to generate *Ppclv1b* single mutants and *Ppclv1a1b* double mutants. An sgRNA was designed against the LRR domain of *PpCLV1b* and expressed under the U6 promoter. Plants were co-transformed with *pU6::Ppclv1b sgRNA*, *pNRF*, and *pACT::Cas9* (original paper). Sanger sequencing of full-length *PpCLV1a* and *PpCLV1b* loci in all lines verified mutations shown in three independently generated lines. Sequences in bold represent sgRNA target sites on WT loci, gray boxes highlight mutations arising, and schematics to the right illustrate the predicted effect of the mutation on the protein. Lines with labels in blue were reported in the original paper. (C) Summary of *PpCLV1* mutations at each locus in nine mutant lines currently in use. Lines with labels in blue were reported in the original paper. (D) Previously described phenotypes of *Ppclv1a*, *Ppclv1b*, and *Ppclv1a1b* mutants are shared by three independently generated lines. Whereas WT plants, *Ppclv1a*, and *Ppclv1b* mutants have well-developed gametophores, *Ppclv1a1b* mutants have a defective 2D-3D growth transition. *Ppclv1b* and *Ppclv1a1b*, but not *Ppclv1a* mutants or WT plants, have tissue outgrowths at the base of gametophores. Lines with labels in bold were included in Figure 4 of the original paper.

Consequently, we have re-engineered and fully sequenced *PpCLV1a* and *PpCLV1b* in three independent *Ppclv1a* and *Ppclv1b* mutant lines to verify the mutant phenotypes reported in Figure 4. The *Ppclv1a* and *Ppclv1b* mutant phenotypes previously reported hold true, and the genotypes and phenotypes of lines now in use are shown in Figure 1 below.

**Figure 2:**
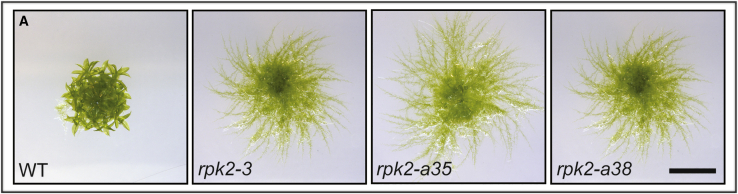
Strategy for Generating *Pprpk2* KO Lines Multiple knockout lines whose genotyping data are shown in Methods S1 *Pprpk2* figure had similar mutant phenotypes.

As we checked our work, we noticed that the genotyping data shown in the Methods S1 figure “Strategy for generating *Pprpk2* KO lines” (Methods S1, *RPK2* figure, panels B–D) were from different lines to those shown in panel E, and we wish to substitute Figure 2 (below) for this panel.

At a late stage of manuscript preparation, the labels for *PpCLV1a* and *PpCLV1b* in Figure S5 and Tables S1 and S3 were transposed from labels elsewhere in the manuscript. The correct identifiers for these genes are as follows:

*PpCLV1a* Pp1s14_447V6.1 (V1.6 genome [2]) and Pp3c6_21940V1.1 (V3 genome [3])
*PpCLV1b* Pp1s5_68V6.1 (V1.6 genome [2]) and Pp3c13_13360V1.1 (V3 genome [3]).

The Lead Contact wishes to apologize to the scientific community for the errors above and any consequent confusion, and thanks Zoe Nemec Venza (University of Bristol), Joe Cammarata (Cornell University), and Wei Liu (University of Bristol) for doing the experimental work required for the correction. We also thank two anonymous referees for commenting on a draft correction notice.

### Method Details

#### Genomic DNA Extraction

Genomic DNA was extracted using a CTAB (hexadecyltrimethylammonium bromide) protocol as described in the original paper.

#### Sequencing

*PpCLV1a* and *PpCLV1b* loci were Sanger sequenced using c. 150 ng template DNA with forward and reverse primers listed in Table 1, below. Sequence traces were analyzed by hand using Chromas Lite software, aligned using BioEdit software, and compared to genome sequence data from the V1.6 *Physcomitrella* genome [2].

#### Genome Walking for Ppclv1b Line 2

A genome walking protocol was adapted from [4]. Genomic DNA was extracted from WT and *Ppclv1b* line 2 plants as above, but with an additional phenol-chloroform-isoamyl alcohol (24:24:1) step following extraction. 1 μg DNA was digested to completion using EcoRV, PvuII, or HpaI and cleaned using a Zymoclean Gel DNA Recovery kit. 20 μL each of 50 mM GenomeWalkerAdaptor1 and GenomeWalkerAdaptor2 adaptors were mixed in water, heated to 100°C for 2 min, and annealed by cooling to room temperature. To generate genome walking libraries, digested genomic DNA was ligated to the annealed adaptors using T4 DNA ligase. To isolate the 3′ junction between *PpCLV1b* and insert sequence, two-step touchdown nested PCR was performed using AP1 + GSP1a primers, and then AP2 + GSP2b primers and Q5 polymerase. To isolate the 5′ junction between *PpCLV1b* and inserted sequence, an M13 primer against the inserted sequence was used with a *PpCLV1b* locus-specific primer, CLV1Blocus_reverse_8. Amplicons were gel purified using a QIAquick PCR Purification kit and sent for direct sequencing.

#### Moss Transformation

New *Ppclv1a* and *Ppclv1b* CRISPR lines were generated as described in the original paper except a single guide RNA expression vector, *U3::Ppclv1a sgRNA7*, was used to generate *Ppclv1a* crA and *Ppclv1a* crB lines.

#### Plant Imaging

Plant imaging was undertaken as described in the original paper.

**Table 1:** List of Genome Walking Primers Used to Prepare the Correction Notice

Primer	Sequence
GenomeWalkerAdaptor1	GTAATACGACTCACTATAGGGCAC GCGTGGTCGACGGCCCGGGCTGGT
GenomeWalkerAdaptor2	3′-H2N-CCCGACCA-PO4-5′
GenomeWalkerAP1	GTAATACGACTCACTATAGGGC
GenomeWalkerAP2	ACTATAGGGCACGCGTGGT
GSP1a	CAGGAGGAATTGGTCCGGTAAGTGAGTT
GSP2b	GCTGGGAAAGATCCATACTGAGAAGGAAT
CLV1Blocus_reverse_8	AAACTCAATCGTCGCAGTGC
M13fw	GTAAAACGACGGCCAG

All primers in 5′-to-3′ orientation unless otherwise stated.

**Table 2:** List of Sequencing Primers Used to Prepare the Correction Notice

Primer	Sequence
CLV1Alocus_forward_1	TGAGCCTGATTGAATCTTAACG
CLV1Alocus_forward_2	AACTCGCTCTCAATGGGCCTCTTC
CLV1Alocus_forward_3	ATCAATCGAATATGTCGTTCCG
CLV1Alocus_forward_4	ATTCCCAGGCTGAGATGAATG
CLV1Alocus_forward_5	ATAGGACCAGAGAGGTTGTTG
CLV1Alocus_forward_6	GATTATCCTGGATCTCTACCATTG
CLV1Alocus_forward_7	CGAGATGATTGTTCATCAAGCTC
CLV1Alocus_forward_8	TCCTCCCAGACTTACGTGTTC
CLV1Alocus_reverse_1	AAAGATGGAGTGCTGGACTTG
CLV1Alocus_reverse_2	AATCCAGGCTGCACATGGTCTTTG
CLV1Alocus_reverse_3	GGTACTTGACTGCTTGGACG
CLV1Alocus_reverse_4	CCGCAATGATGGTGCTCCTTGTAG
CLV1Alocus_reverse_5	ATGAGCGGGAACAATTTATCAG
CLV1Alocus_reverse_6	ATCCTCTGAATCCAATGCCG
CLV1Alocus_reverse_7	CTGGTTGGAGCAATCCCACATGAG
CLV1Alocus_reverse_8	GATAACTTGTCTGAAGCCCATC
CLV1Alocus_reverse_9	GGCCGAAGTGAGGTACATATTTAG
CLV1Blocus_forward_1	CTGAGTGAGAAGAGTGACACATC
CLV1Blocus_forward_2	ACGTCGAGTCTCTACGCAAC
CLV1Blocus_forward_3	CCCTTTATACACAGTTCCAGC
CLV1Blocus_forward_4	GTATGAGAAGTCGAATACGTTGAG
CLV1Blocus_forward_5	GATCGACCCATTCAGAAGATTG
CLV1Blocus_forward_6	TGCAGGAACATGGAGTCGAGATTG
CLV1Blocus_forward_7	CGTGTGCATTACTTCCTGTGTTG
CLV1Blocus_forward_8	TCTTCACCATTCTTGCTTCTG
CLV1Blocus_forward_9	TGTTACTGCGAAGTGTGCTA
CLV1Blocus_reverse_1	TTAGGGTGGTGCATGAACTGCTTG
CLV1Blocus_reverse_2	CGGATTCTCAGCAGAGATTCAAAC
CLV1Blocus_reverse_3	AATAACCTCTCAGGACCAATTCC
CLV1Blocus_reverse_4	CGTTGGGTTTGCTTGGCTTG
CLV1Blocus_reverse_5	ATGAACTCGTAGGTGTCATCCC
CLV1Blocus_reverse_6	CAGGAAGTAATGCACACGCC
CLV1Blocus_reverse_7	ACGCAACTTCCATATAGTCTCTGC
CLV1Blocus_reverse_7a	GGAGAGACGCAACTTCCATATAG
CLV1Blocus_reverse_8	AAACTCAATCGTCGCAGTGC
